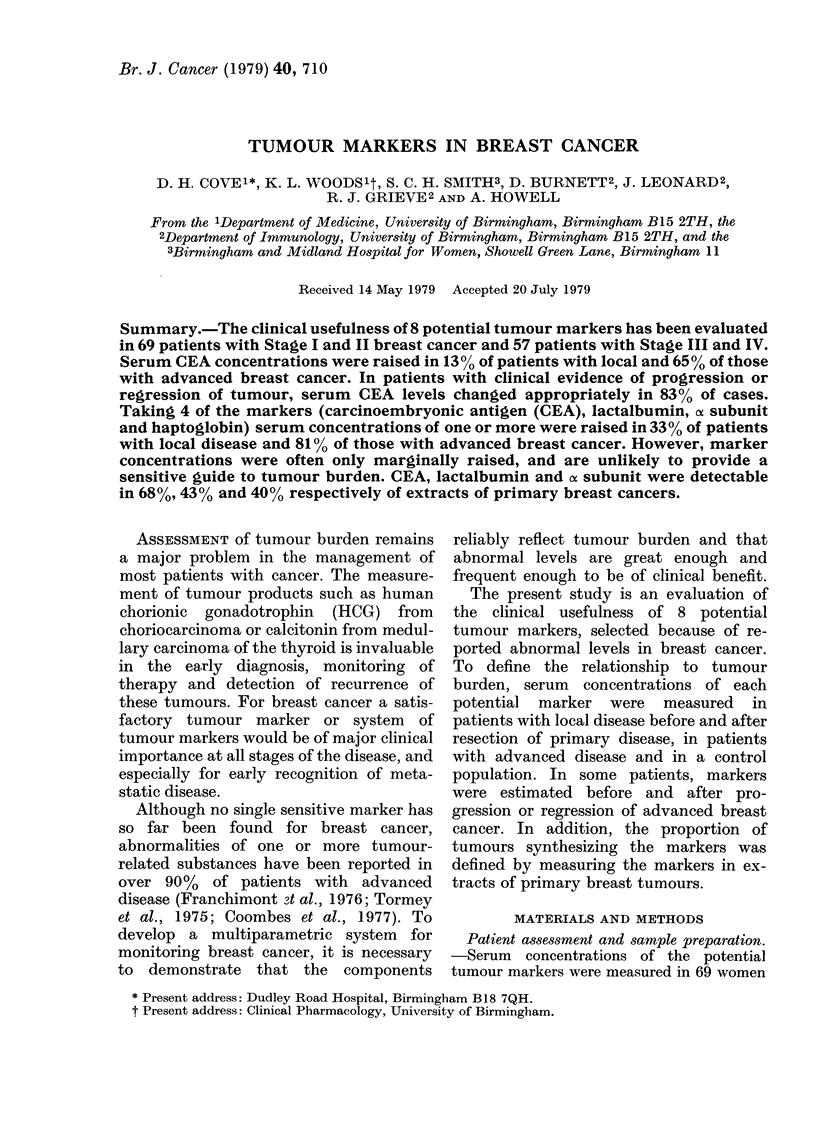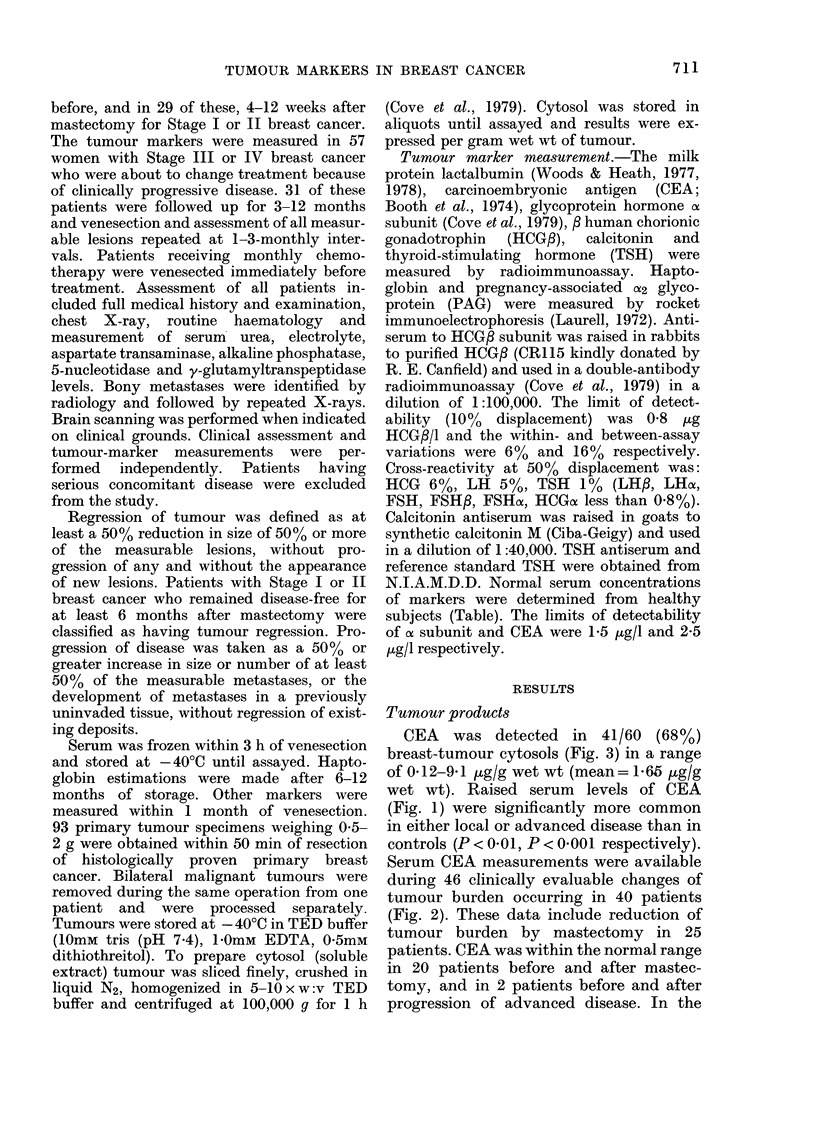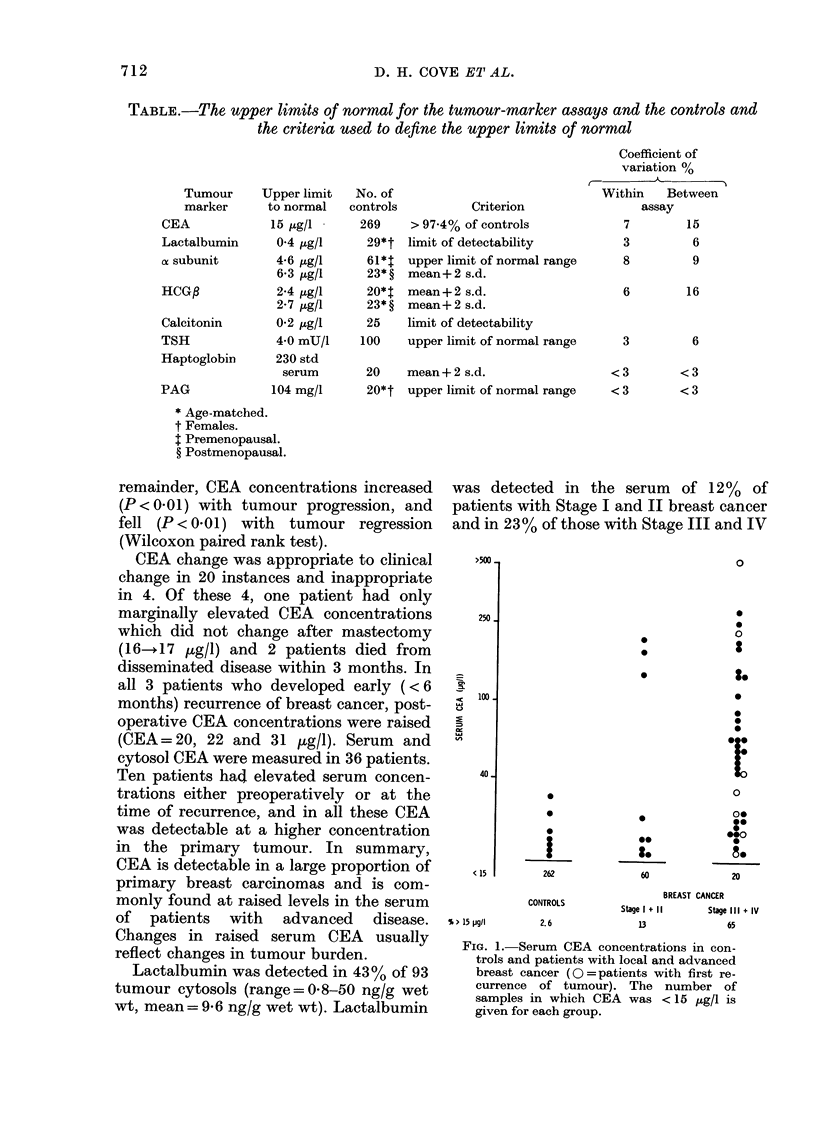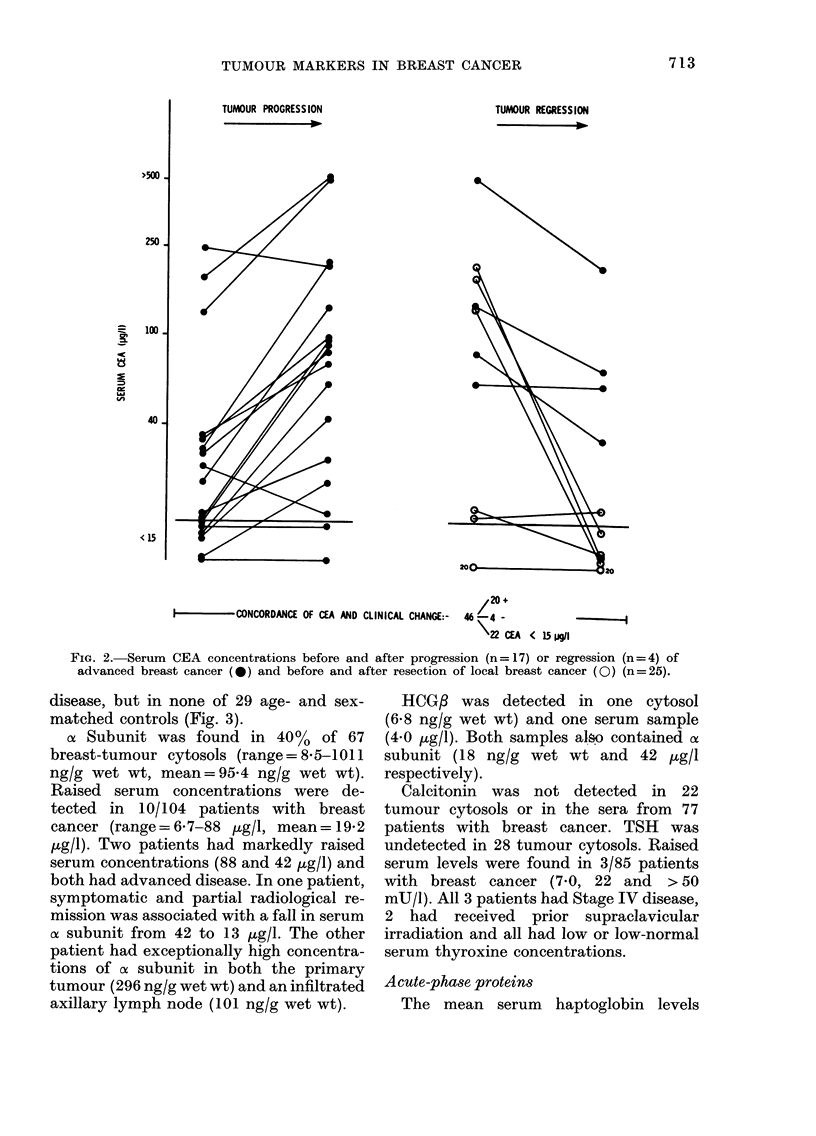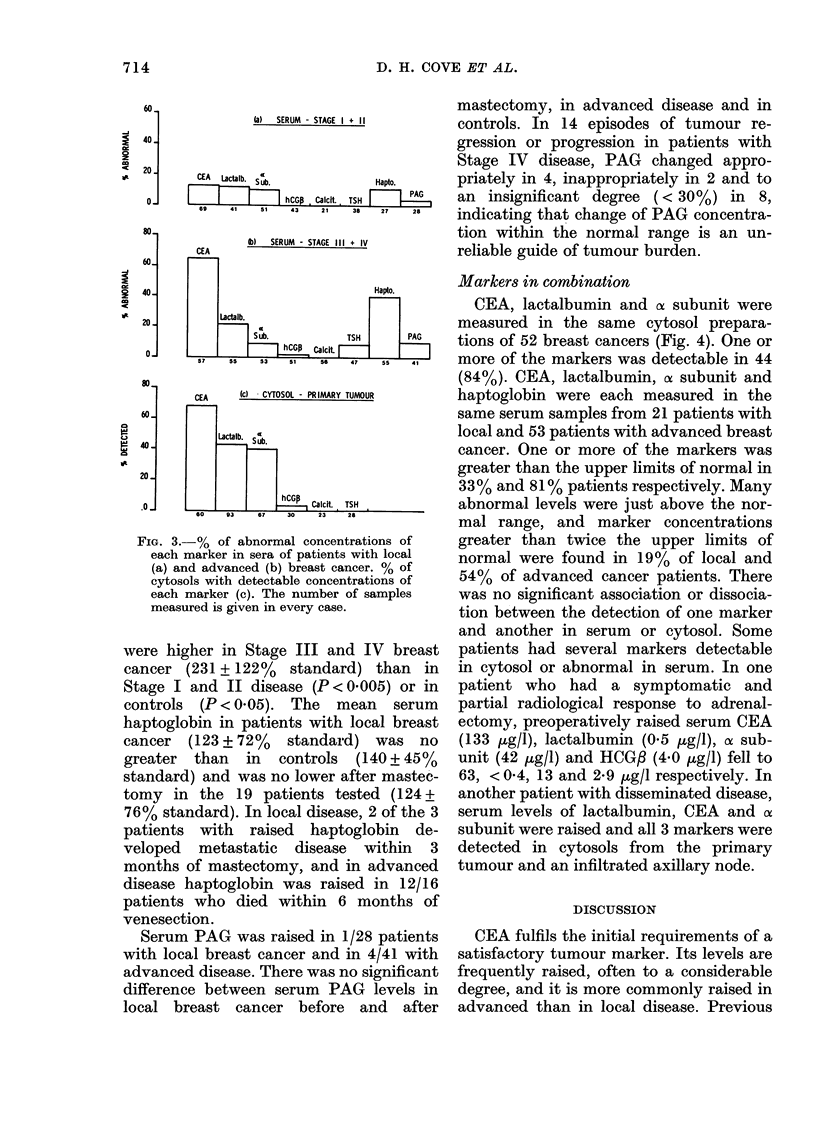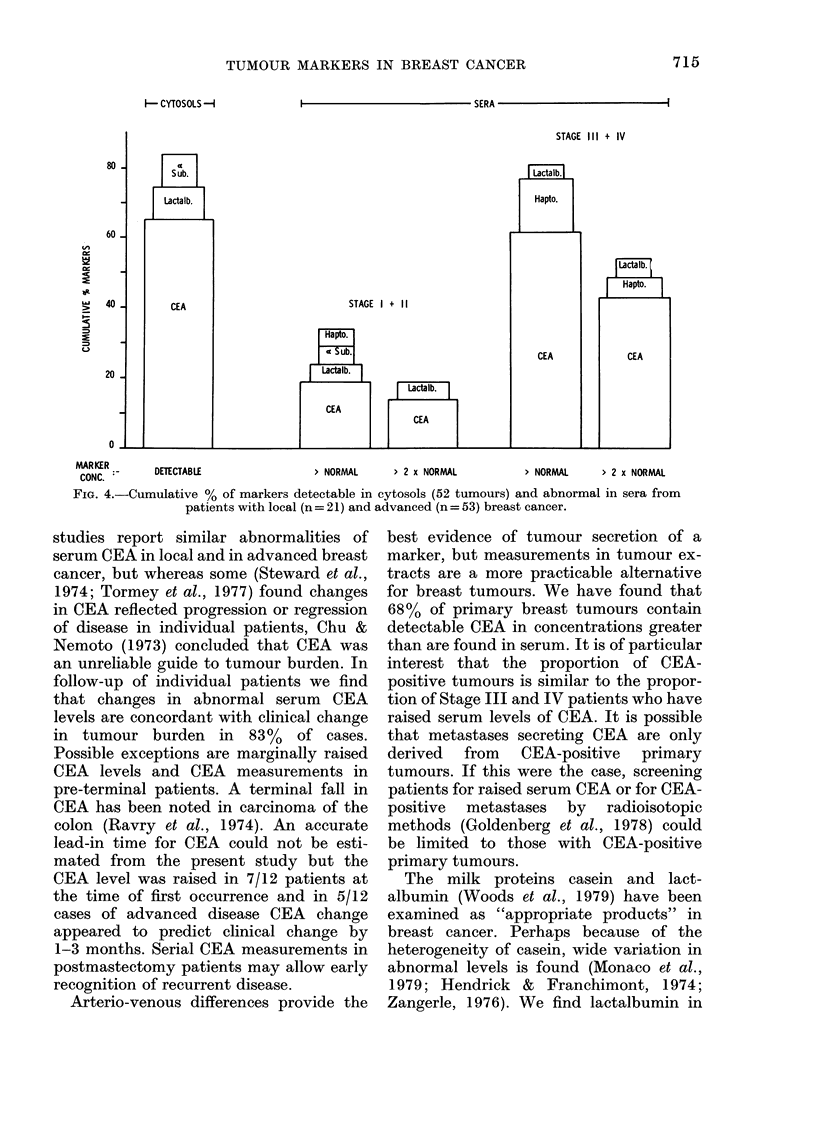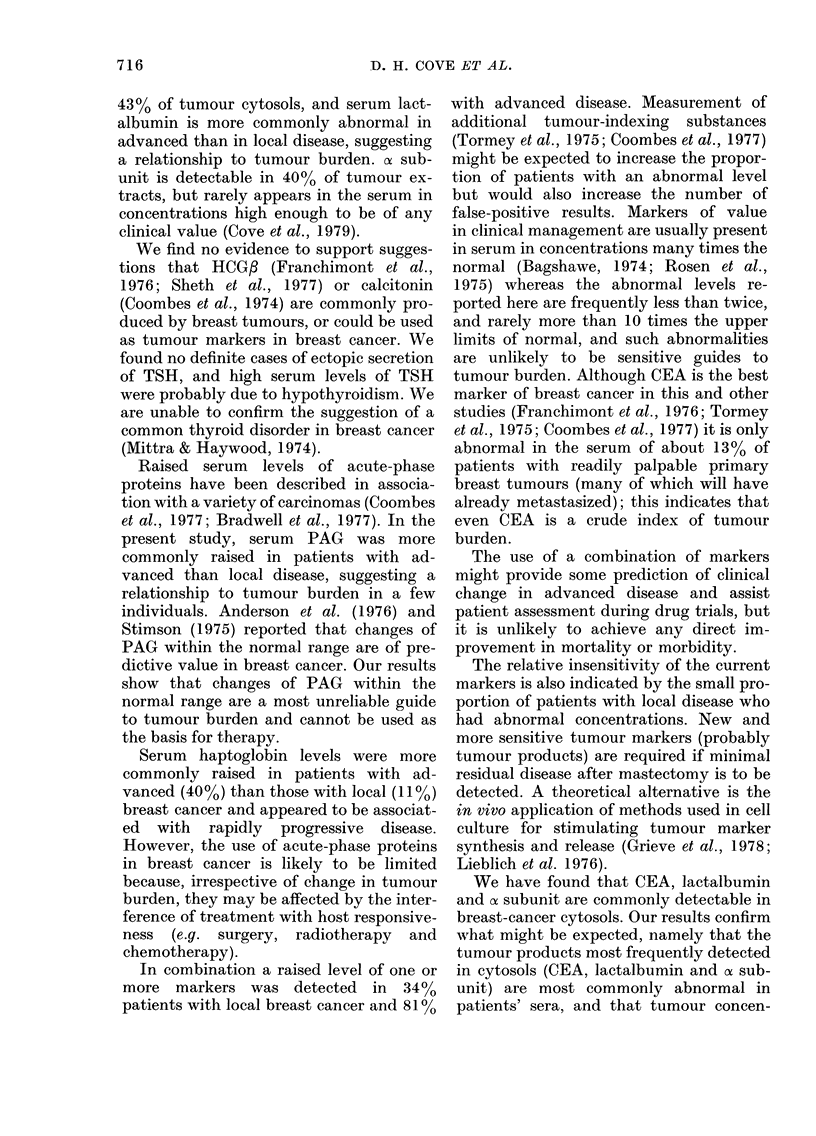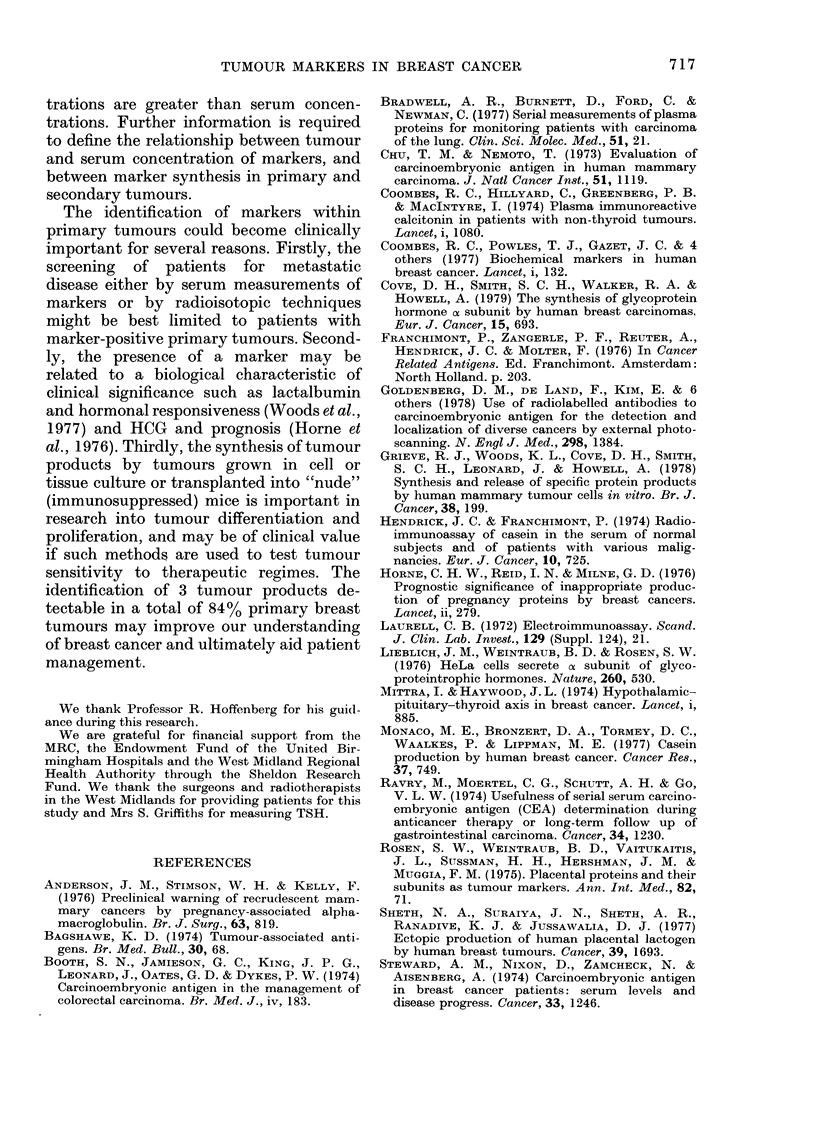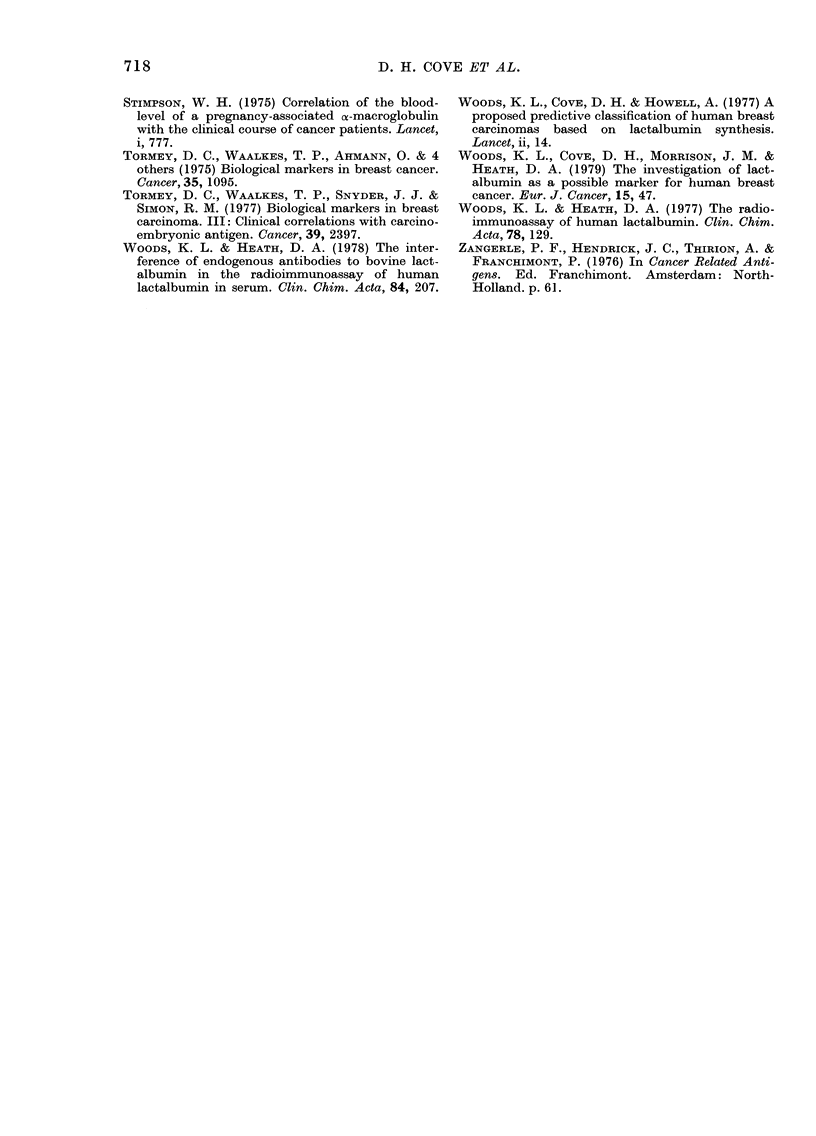# Tumour markers in breast cancer.

**DOI:** 10.1038/bjc.1979.251

**Published:** 1979-11

**Authors:** D. H. Cove, K. L. Woods, S. C. Smith, D. Burnett, J. Leonard, R. J. Grieve, A. Howell

## Abstract

The clinical usefulness of 8 potential tumour markers has been evaluated in 69 patients with Stage I and II breast cancer and 57 patients with Stage III and IV. Serum CEA concentrations were raised in 13% of patients with local and 65% of those with advanced breast cancer. In patients with clinical evidence of progression or regression of tumour, serum CEA levels changed appropriately in 83% of cases. Taking 4 of the markers (carcinoembryonic antigen (CEA), lactalbumin, alpha subunit and haptoglobin) serum concentrations of one or more were raised in 33% of patients with local disease and 81% of those with advanced breast cancer. However, marker concentrations were often only marginally raised, and are unlikely to provide sensitive guide to tumour burden. CEA, lactalbumin and alpha subunit were detectable in 68%, 43% and 40% respectively of extracts of primary breast cancers.


*Br. J. Cancer* (1979) **40**, 710

### TUMOUR MARKERS IN BREAST CANCER

D. H. COVE[1*], K. L. WOODS[1†], S. C. H. SMITH[3], D. BURNETT[2], J. LEONARD[2], R. J. GRIEVE[2] AND A. HOWELL

*From the [1]Department of Medicine, University of Birmingham, Birmingham B15 2TH, the [2]Department of Immunology, University of Birmingham, Birmingham B15 2TH, and the [3]Birmingham and Midland Hospital for Women, Showell Green Lane, Birmingham 11*

Received 14 May 1979 Accepted 20 July 1979

**Summary.—The clinical usefulness of 8 potential tumour markers has been evaluated in 69 patients with Stage I and II breast cancer and 57 patients with Stage III and IV. Serum CEA concentrations were raised in 13% of patients with local and 65% of those with advanced breast cancer. In patients with clinical evidence of progression or regression of tumour, serum CEA levels changed appropriately in 83% of cases. Taking 4 of the markers (carcinoembryonic antigen (CEA), lactalbumin, α subunit and haptoglobin) serum concentrations of one or more were raised in 33% of patients with local disease and 81% of those with advanced breast cancer. However, marker concentrations were often only marginally raised, and are unlikely to provide a sensitive guide to tumour burden. CEA, lactalbumin and α subunit were detectable in 68%, 43% and 40% respectively of extracts of primary breast cancers.**

ASSESSMENT of tumour burden remains a major problem in the management of most patients with cancer. The measurement of tumour products such as human chorionic gonadotrophin (HCG) from choriocarcinoma or calcitonin from medullary carcinoma of the thyroid is invaluable in the early diagnosis, monitoring of therapy and detection of recurrence of these tumours. For breast cancer a satisfactory tumour marker or system of tumour markers would be of major clinical importance at all stages of the disease, and especially for early recognition of metastatic disease.

Although no single sensitive marker has so far been found for breast cancer, abnormalities of one or more tumour-related substances have been reported in over 90% of patients with advanced disease (Franchimont *et al.*, 1976; Tormey *et al.*, 1975; Coombes *et al.*, 1977). To develop a multiparametric system for monitoring breast cancer, it is necessary to demonstrate that the components reliably reflect tumour burden and that abnormal levels are great enough and frequent enough to be of clinical benefit.

The present study is an evaluation of the clinical usefulness of 8 potential tumour markers, selected because of reported abnormal levels in breast cancer. To define the relationship to tumour burden, serum concentrations of each potential marker were measured in patients with local disease before and after resection of primary disease, in patients with advanced disease and in a control population. In some patients, markers were estimated before and after progression or regression of advanced breast cancer. In addition, the proportion of tumours synthesizing the markers was defined by measuring the markers in extracts of primary breast tumours.

### MATERIALS AND METHODS

*Patient assessment and sample preparation.*—Serum concentrations of the potential tumour markers were measured in 69 women

* Present address: Dudley Road Hospital, Birmingham B18 7QH.
† Present address: Clinical Pharmacology, University of Birmingham.

before, and in 29 of these, 4–12 weeks after mastectomy for Stage I or II breast cancer. The tumour markers were measured in 57 women with Stage III or IV breast cancer who were about to change treatment because of clinically progressive disease. 31 of these patients were followed up for 3–12 months and venesection and assessment of all measurable lesions repeated at 1–3-monthly intervals. Patients receiving monthly chemotherapy were venesected immediately before treatment. Assessment of all patients included full medical history and examination, chest X-ray, routine haematology and measurement of serum urea, electrolyte, aspartate transaminase, alkaline phosphatase, 5-nucleotidase and $\gamma$-glutamyltranspeptidase levels. Bony metastases were identified by radiology and followed by repeated X-rays. Brain scanning was performed when indicated on clinical grounds. Clinical assessment and tumour-marker measurements were performed independently. Patients having serious concomitant disease were excluded from the study.

Regression of tumour was defined as at least a 50% reduction in size of 50% or more of the measurable lesions, without progression of any and without the appearance of new lesions. Patients with Stage I or II breast cancer who remained disease-free for at least 6 months after mastectomy were classified as having tumour regression. Progression of disease was taken as a 50% or greater increase in size or number of at least 50% of the measurable metastases, or the development of metastases in a previously uninvaded tissue, without regression of existing deposits.

Serum was frozen within 3 h of venesection and stored at $-40$°C until assayed. Haptoglobin estimations were made after 6–12 months of storage. Other markers were measured within 1 month of venesection. 93 primary tumour specimens weighing 0·5–2 g were obtained within 50 min of resection of histologically proven primary breast cancer. Bilateral malignant tumours were removed during the same operation from one patient and were processed separately. Tumours were stored at $-40$°C in TED buffer (10mM tris (pH 7·4), 1·0mM EDTA, 0·5mM dithiothreitol). To prepare cytosol (soluble extract) tumour was sliced finely, crushed in liquid $N_2$, homogenized in 5–10 × w:v TED buffer and centrifuged at 100,000 *g* for 1 h (Cove *et al.*, 1979). Cytosol was stored in aliquots until assayed and results were expressed per gram wet wt of tumour.

*Tumour marker measurement.*—The milk protein lactalbumin (Woods & Heath, 1977, 1978), carcinoembryonic antigen (CEA; Booth *et al.*, 1974), glycoprotein hormone $\alpha$ subunit (Cove *et al.*, 1979), $\beta$ human chorionic gonadotrophin (HCG$\beta$), calcitonin and thyroid-stimulating hormone (TSH) were measured by radioimmunoassay. Haptoglobin and pregnancy-associated $\alpha_2$ glycoprotein (PAG) were measured by rocket immunoelectrophoresis (Laurell, 1972). Antiserum to HCG$\beta$ subunit was raised in rabbits to purified HCG$\beta$ (CR115 kindly donated by R. E. Canfield) and used in a double-antibody radioimmunoassay (Cove *et al.*, 1979) in a dilution of 1:100,000. The limit of detectability (10% displacement) was 0·8 $\mu$g HCG$\beta$/l and the within- and between-assay variations were 6% and 16% respectively. Cross-reactivity at 50% displacement was: HCG 6%, LH 5%, TSH 1% (LH$\beta$, LH$\alpha$, FSH, FSH$\beta$, FSH$\alpha$, HCG$\alpha$ less than 0·8%). Calcitonin antiserum was raised in goats to synthetic calcitonin M (Ciba-Geigy) and used in a dilution of 1:40,000. TSH antiserum and reference standard TSH were obtained from N.I.A.M.D.D. Normal serum concentrations of markers were determined from healthy subjects (Table). The limits of detectability of $\alpha$ subunit and CEA were 1·5 $\mu$g/l and 2·5 $\mu$g/l respectively.

### RESULTS

### *Tumour products*

CEA was detected in 41/60 (68%) breast-tumour cytosols (Fig. 3) in a range of 0·12–9·1 $\mu$g/g wet wt (mean = 1·65 $\mu$g/g wet wt). Raised serum levels of CEA (Fig. 1) were significantly more common in either local or advanced disease than in controls ($P < 0{\cdot}01$, $P < 0{\cdot}001$ respectively). Serum CEA measurements were available during 46 clinically evaluable changes of tumour burden occurring in 40 patients (Fig. 2). These data include reduction of tumour burden by mastectomy in 25 patients. CEA was within the normal range in 20 patients before and after mastectomy, and in 2 patients before and after progression of advanced disease. In the

TABLE.—*The upper limits of normal for the tumour-marker assays and the controls and the criteria used to define the upper limits of normal*

| Tumour marker | Upper limit to normal | No. of controls | Criterion | Coefficient of variation % Within assay | Between assay |
|---|---|---|---|---|---|
| CEA | 15 μg/l | 269 | > 97·4% of controls | 7 | 15 |
| Lactalbumin | 0·4 μg/l | 29*† | limit of detectability | 3 | 6 |
| α subunit | 4·6 μg/l | 61*‡ | upper limit of normal range | 8 | 9 |
| | 6·3 μg/l | 23*§ | mean + 2 s.d. | | |
| HCGβ | 2·4 μg/l | 20*‡ | mean + 2 s.d. | 6 | 16 |
| | 2·7 μg/l | 23*§ | mean + 2 s.d. | | |
| Calcitonin | 0·2 μg/l | 25 | limit of detectability | | |
| TSH | 4·0 mU/l | 100 | upper limit of normal range | 3 | 6 |
| Haptoglobin | 230 std serum | 20 | mean + 2 s.d. | < 3 | < 3 |
| PAG | 104 mg/l | 20*† | upper limit of normal range | < 3 | < 3 |

* Age-matched.
† Females.
‡ Premenopausal.
§ Postmenopausal.

remainder, CEA concentrations increased ($P < 0·01$) with tumour progression, and fell ($P < 0·01$) with tumour regression (Wilcoxon paired rank test).

CEA change was appropriate to clinical change in 20 instances and inappropriate in 4. Of these 4, one patient had only marginally elevated CEA concentrations which did not change after mastectomy (16→17 μg/l) and 2 patients died from disseminated disease within 3 months. In all 3 patients who developed early (< 6 months) recurrence of breast cancer, postoperative CEA concentrations were raised (CEA = 20, 22 and 31 μg/l). Serum and cytosol CEA were measured in 36 patients. Ten patients had elevated serum concentrations either preoperatively or at the time of recurrence, and in all these CEA was detectable at a higher concentration in the primary tumour. In summary, CEA is detectable in a large proportion of primary breast carcinomas and is commonly found at raised levels in the serum of patients with advanced disease. Changes in raised serum CEA usually reflect changes in tumour burden.

Lactalbumin was detected in 43% of 93 tumour cytosols (range = 0·8–50 ng/g wet wt, mean = 9·6 ng/g wet wt). Lactalbumin was detected in the serum of 12% of patients with Stage I and II breast cancer and in 23% of those with Stage III and IV

FIG. 1.—Serum CEA concentrations in controls and patients with local and advanced breast cancer (○ = patients with first recurrence of tumour). The number of samples in which CEA was < 15 μg/l is given for each group.

FIG. 2.—Serum CEA concentrations before and after progression (n = 17) or regression (n = 4) of advanced breast cancer (●) and before and after resection of local breast cancer (○) (n = 25).

disease, but in none of 29 age- and sex-matched controls (Fig. 3).

α Subunit was found in 40% of 67 breast-tumour cytosols (range = 8·5–1011 ng/g wet wt, mean = 95·4 ng/g wet wt). Raised serum concentrations were detected in 10/104 patients with breast cancer (range = 6·7–88 µg/l, mean = 19·2 µg/l). Two patients had markedly raised serum concentrations (88 and 42 µg/l) and both had advanced disease. In one patient, symptomatic and partial radiological remission was associated with a fall in serum α subunit from 42 to 13 µg/l. The other patient had exceptionally high concentrations of α subunit in both the primary tumour (296 ng/g wet wt) and an infiltrated axillary lymph node (101 ng/g wet wt).

HCGβ was detected in one cytosol (6·8 ng/g wet wt) and one serum sample (4·0 µg/l). Both samples also contained α subunit (18 ng/g wet wt and 42 µg/l respectively).

Calcitonin was not detected in 22 tumour cytosols or in the sera from 77 patients with breast cancer. TSH was undetected in 28 tumour cytosols. Raised serum levels were found in 3/85 patients with breast cancer (7·0, 22 and > 50 mU/l). All 3 patients had Stage IV disease, 2 had received prior supraclavicular irradiation and all had low or low-normal serum thyroxine concentrations.

*Acute-phase proteins*

The mean serum haptoglobin levels

FIG. 3.—% of abnormal concentrations of each marker in sera of patients with local (a) and advanced (b) breast cancer. % of cytosols with detectable concentrations of each marker (c). The number of samples measured is given in every case.

were higher in Stage III and IV breast cancer (231 ± 122% standard) than in Stage I and II disease ($P < 0{\cdot}005$) or in controls ($P < 0{\cdot}05$). The mean serum haptoglobin in patients with local breast cancer (123 ± 72% standard) was no greater than in controls (140 ± 45% standard) and was no lower after mastectomy in the 19 patients tested (124 ± 76% standard). In local disease, 2 of the 3 patients with raised haptoglobin developed metastatic disease within 3 months of mastectomy, and in advanced disease haptoglobin was raised in 12/16 patients who died within 6 months of venesection.

Serum PAG was raised in 1/28 patients with local breast cancer and in 4/41 with advanced disease. There was no significant difference between serum PAG levels in local breast cancer before and after mastectomy, in advanced disease and in controls. In 14 episodes of tumour regression or progression in patients with Stage IV disease, PAG changed appropriately in 4, inappropriately in 2 and to an insignificant degree (< 30%) in 8, indicating that change of PAG concentration within the normal range is an unreliable guide of tumour burden.

*Markers in combination*

CEA, lactalbumin and α subunit were measured in the same cytosol preparations of 52 breast cancers (Fig. 4). One or more of the markers was detectable in 44 (84%). CEA, lactalbumin, α subunit and haptoglobin were each measured in the same serum samples from 21 patients with local and 53 patients with advanced breast cancer. One or more of the markers was greater than the upper limits of normal in 33% and 81% patients respectively. Many abnormal levels were just above the normal range, and marker concentrations greater than twice the upper limits of normal were found in 19% of local and 54% of advanced cancer patients. There was no significant association or dissociation between the detection of one marker and another in serum or cytosol. Some patients had several markers detectable in cytosol or abnormal in serum. In one patient who had a symptomatic and partial radiological response to adrenalectomy, preoperatively raised serum CEA (133 μg/l), lactalbumin (0·5 μg/l), α subunit (42 μg/l) and HCGβ (4·0 μg/l) fell to 63, < 0·4, 13 and 2·9 μg/l respectively. In another patient with disseminated disease, serum levels of lactalbumin, CEA and α subunit were raised and all 3 markers were detected in cytosols from the primary tumour and an infiltrated axillary node.

### DISCUSSION

CEA fulfils the initial requirements of a satisfactory tumour marker. Its levels are frequently raised, often to a considerable degree, and it is more commonly raised in advanced than in local disease. Previous

FIG. 4.—Cumulative % of markers detectable in cytosols (52 tumours) and abnormal in sera from patients with local (n = 21) and advanced (n = 53) breast cancer.

studies report similar abnormalities of serum CEA in local and in advanced breast cancer, but whereas some (Steward *et al.*, 1974; Tormey *et al.*, 1977) found changes in CEA reflected progression or regression of disease in individual patients, Chu & Nemoto (1973) concluded that CEA was an unreliable guide to tumour burden. In follow-up of individual patients we find that changes in abnormal serum CEA levels are concordant with clinical change in tumour burden in 83% of cases. Possible exceptions are marginally raised CEA levels and CEA measurements in pre-terminal patients. A terminal fall in CEA has been noted in carcinoma of the colon (Ravry *et al.*, 1974). An accurate lead-in time for CEA could not be estimated from the present study but the CEA level was raised in 7/12 patients at the time of first occurrence and in 5/12 cases of advanced disease CEA change appeared to predict clinical change by 1–3 months. Serial CEA measurements in postmastectomy patients may allow early recognition of recurrent disease.

Arterio-venous differences provide the best evidence of tumour secretion of a marker, but measurements in tumour extracts are a more practicable alternative for breast tumours. We have found that 68% of primary breast tumours contain detectable CEA in concentrations greater than are found in serum. It is of particular interest that the proportion of CEA-positive tumours is similar to the proportion of Stage III and IV patients who have raised serum levels of CEA. It is possible that metastases secreting CEA are only derived from CEA-positive primary tumours. If this were the case, screening patients for raised serum CEA or for CEA-positive metastases by radioisotopic methods (Goldenberg *et al.*, 1978) could be limited to those with CEA-positive primary tumours.

The milk proteins casein and lactalbumin (Woods *et al.*, 1979) have been examined as "appropriate products" in breast cancer. Perhaps because of the heterogeneity of casein, wide variation in abnormal levels is found (Monaco *et al.*, 1979; Hendrick & Franchimont, 1974; Zangerle, 1976). We find lactalbumin in

43% of tumour cytosols, and serum lactalbumin is more commonly abnormal in advanced than in local disease, suggesting a relationship to tumour burden. $\alpha$ subunit is detectable in 40% of tumour extracts, but rarely appears in the serum in concentrations high enough to be of any clinical value (Cove *et al.*, 1979).

We find no evidence to support suggestions that HCG$\beta$ (Franchimont *et al.*, 1976; Sheth *et al.*, 1977) or calcitonin (Coombes *et al.*, 1974) are commonly produced by breast tumours, or could be used as tumour markers in breast cancer. We found no definite cases of ectopic secretion of TSH, and high serum levels of TSH were probably due to hypothyroidism. We are unable to confirm the suggestion of a common thyroid disorder in breast cancer (Mittra & Haywood, 1974).

Raised serum levels of acute-phase proteins have been described in association with a variety of carcinomas (Coombes *et al.*, 1977; Bradwell *et al.*, 1977). In the present study, serum PAG was more commonly raised in patients with advanced than local disease, suggesting a relationship to tumour burden in a few individuals. Anderson *et al.* (1976) and Stimson (1975) reported that changes of PAG within the normal range are of predictive value in breast cancer. Our results show that changes of PAG within the normal range are a most unreliable guide to tumour burden and cannot be used as the basis for therapy.

Serum haptoglobin levels were more commonly raised in patients with advanced (40%) than those with local (11%) breast cancer and appeared to be associated with rapidly progressive disease. However, the use of acute-phase proteins in breast cancer is likely to be limited because, irrespective of change in tumour burden, they may be affected by the interference of treatment with host responsiveness (*e.g.* surgery, radiotherapy and chemotherapy).

In combination a raised level of one or more markers was detected in 34% patients with local breast cancer and 81% with advanced disease. Measurement of additional tumour-indexing substances (Tormey *et al.*, 1975; Coombes *et al.*, 1977) might be expected to increase the proportion of patients with an abnormal level but would also increase the number of false-positive results. Markers of value in clinical management are usually present in serum in concentrations many times the normal (Bagshawe, 1974; Rosen *et al.*, 1975) whereas the abnormal levels reported here are frequently less than twice, and rarely more than 10 times the upper limits of normal, and such abnormalities are unlikely to be sensitive guides to tumour burden. Although CEA is the best marker of breast cancer in this and other studies (Franchimont *et al.*, 1976; Tormey *et al.*, 1975; Coombes *et al.*, 1977) it is only abnormal in the serum of about 13% of patients with readily palpable primary breast tumours (many of which will have already metastasized); this indicates that even CEA is a crude index of tumour burden.

The use of a combination of markers might provide some prediction of clinical change in advanced disease and assist patient assessment during drug trials, but it is unlikely to achieve any direct improvement in mortality or morbidity.

The relative insensitivity of the current markers is also indicated by the small proportion of patients with local disease who had abnormal concentrations. New and more sensitive tumour markers (probably tumour products) are required if minimal residual disease after mastectomy is to be detected. A theoretical alternative is the *in vivo* application of methods used in cell culture for stimulating tumour marker synthesis and release (Grieve *et al.*, 1978; Lieblich *et al.* 1976).

We have found that CEA, lactalbumin and $\alpha$ subunit are commonly detectable in breast-cancer cytosols. Our results confirm what might be expected, namely that the tumour products most frequently detected in cytosols (CEA, lactalbumin and $\alpha$ subunit) are most commonly abnormal in patients' sera, and that tumour concentrations are greater than serum concentrations. Further information is required to define the relationship between tumour and serum concentration of markers, and between marker synthesis in primary and secondary tumours.

The identification of markers within primary tumours could become clinically important for several reasons. Firstly, the screening of patients for metastatic disease either by serum measurements of markers or by radioisotopic techniques might be best limited to patients with marker-positive primary tumours. Secondly, the presence of a marker may be related to a biological characteristic of clinical significance such as lactalbumin and hormonal responsiveness (Woods *et al.*, 1977) and HCG and prognosis (Horne *et al.*, 1976). Thirdly, the synthesis of tumour products by tumours grown in cell or tissue culture or transplanted into "nude" (immunosuppressed) mice is important in research into tumour differentiation and proliferation, and may be of clinical value if such methods are used to test tumour sensitivity to therapeutic regimes. The identification of 3 tumour products detectable in a total of 84% primary breast tumours may improve our understanding of breast cancer and ultimately aid patient management.

We thank Professor R. Hoffenberg for his guidance during this research.

We are grateful for financial support from the MRC, the Endowment Fund of the United Birmingham Hospitals and the West Midland Regional Health Authority through the Sheldon Research Fund. We thank the surgeons and radiotherapists in the West Midlands for providing patients for this study and Mrs S. Griffiths for measuring TSH.

### REFERENCES

ANDERSON, J. M., STIMSON, W. H. & KELLY, F. (1976) Preclinical warning of recrudescent mammary cancers by pregnancy-associated alphamacroglobulin. *Br. J. Surg.*, **63**, 819.

BAGSHAWE, K. D. (1974) Tumour-associated antigens. *Br. Med. Bull.*, **30**, 68.

BOOTH, S. N., JAMIESON, G. C., KING, J. P. G., LEONARD, J., OATES, G. D. & DYKES, P. W. (1974) Carcinoembryonic antigen in the management of colorectal carcinoma. *Br. Med. J.*, iv, 183.

BRADWELL, A. R., BURNETT, D., FORD, C. & NEWMAN, C. (1977) Serial measurements of plasma proteins for monitoring patients with carcinoma of the lung. *Clin. Sci. Molec. Med.*, **51**, 21.

CHU, T. M. & NEMOTO, T. (1973) Evaluation of carcinoembryonic antigen in human mammary carcinoma. *J. Natl Cancer Inst.*, **51**, 1119.

COOMBES, R. C., HILLYARD, C., GREENBERG, P. B. & MACINTYRE, I. (1974) Plasma immunoreactive calcitonin in patients with non-thyroid tumours. *Lancet*, i, 1080.

COOMBES, R. C., POWLES, T. J., GAZET, J. C. & 4 others (1977) Biochemical markers in human breast cancer. *Lancet*, i, 132.

COVE, D. H., SMITH, S. C. H., WALKER, R. A. & HOWELL, A. (1979) The synthesis of glycoprotein hormone α subunit by human breast carcinomas. *Eur. J. Cancer*, **15**, 693.

FRANCHIMONT, P., ZANGERLE, P. F., REUTER, A., HENDRICK, J. C. & MOLTER, F. (1976) In *Cancer Related Antigens*. Ed. Franchimont. Amsterdam: North Holland. p. 203.

GOLDENBERG, D. M., DE LAND, F., KIM, E. & 6 others (1978) Use of radiolabelled antibodies to carcinoembryonic antigen for the detection and localization of diverse cancers by external photoscanning. *N. Engl J. Med.*, **298**, 1384.

GRIEVE, R. J., WOODS, K. L., COVE, D. H., SMITH, S. C. H., LEONARD, J. & HOWELL, A. (1978) Synthesis and release of specific protein products by human mammary tumour cells *in vitro*. *Br. J. Cancer*, **38**, 199.

HENDRICK, J. C. & FRANCHIMONT, P. (1974) Radioimmunoassay of casein in the serum of normal subjects and of patients with various malignancies. *Eur. J. Cancer*, **10**, 725.

HORNE, C. H. W., REID, I. N. & MILNE, G. D. (1976) Prognostic significance of inappropriate production of pregnancy proteins by breast cancers. *Lancet*, ii, 279.

LAURELL, C. B. (1972) Electroimmunoassay. *Scand. J. Clin. Lab. Invest.*, **129** (Suppl. 124), 21.

LIEBLICH, J. M., WEINTRAUB, B. D. & ROSEN, S. W. (1976) HeLa cells secrete α subunit of glycoproteintrophic hormones. *Nature*, **260**, 530.

MITTRA, I. & HAYWOOD, J. L. (1974) Hypothalamic–pituitary–thyroid axis in breast cancer. *Lancet*, i, 885.

MONACO, M. E., BRONZERT, D. A., TORMEY, D. C., WAALKES, P. & LIPPMAN, M. E. (1977) Casein production by human breast cancer. *Cancer Res.*, **37**, 749.

RAVRY, M., MOERTEL, C. G., SCHUTT, A. H. & GO, V. L. W. (1974) Usefulness of serial serum carcinoembryonic antigen (CEA) determination during anticancer therapy or long-term follow up of gastrointestinal carcinoma. *Cancer*, **34**, 1230.

ROSEN, S. W., WEINTRAUB, B. D., VAITUKAITIS, J. L., SUSSMAN, H. H., HERSHMAN, J. M. & MUGGIA, F. M. (1975). Placental proteins and their subunits as tumour markers. *Ann. Int. Med.*, **82**, 71.

SHETH, N. A., SURAIYA, J. N., SHETH, A. R., RANADIVE, K. J. & JUSSAWALIA, D. J. (1977) Ectopic production of human placental lactogen by human breast tumours. *Cancer*, **39**, 1693.

STEWARD, A. M., NIXON, D., ZAMCHECK, N. & AISENBERG, A. (1974) Carcinoembryonic antigen in breast cancer patients: serum levels and disease progress. *Cancer*, **33**, 1246.

STIMPSON, W. H. (1975) Correlation of the blood-level of a pregnancy-associated $\alpha$-macroglobulin with the clinical course of cancer patients. *Lancet*, i, 777.

TORMEY, D. C., WAALKES, T. P., AHMANN, O. & 4 others (1975) Biological markers in breast cancer. *Cancer*, **35**, 1095.

TORMEY, D. C., WAALKES, T. P., SNYDER, J. J. & SIMON, R. M. (1977) Biological markers in breast carcinoma. III: Clinical correlations with carcinoembryonic antigen. *Cancer*, **39**, 2397.

WOODS, K. L. & HEATH, D. A. (1978) The interference of endogenous antibodies to bovine lactalbumin in the radioimmunoassay of human lactalbumin in serum. *Clin. Chim. Acta*, **84**, 207.

WOODS, K. L., COVE, D. H. & HOWELL, A. (1977) A proposed predictive classification of human breast carcinomas based on lactalbumin synthesis. *Lancet*, ii, 14.

WOODS, K. L., COVE, D. H., MORRISON, J. M. & HEATH, D. A. (1979) The investigation of lactalbumin as a possible marker for human breast cancer. *Eur. J. Cancer*, **15**, 47.

WOODS, K. L. & HEATH, D. A. (1977) The radioimmunoassay of human lactalbumin. *Clin. Chim. Acta*, **78**, 129.

ZANGERLE, P. F., HENDRICK, J. C., THIRION, A. & FRANCHIMONT, P. (1976) In *Cancer Related Antigens*. Ed. Franchimont. Amsterdam: North-Holland. p. 61.